# Association Between Weight Status and Executive Function in Young Adults

**DOI:** 10.3390/medicina55070363

**Published:** 2019-07-10

**Authors:** Mohammad Narimani, Samad Esmaeilzadeh, Liane B. Azevedo, Akbar Moradi, Behrouz Heidari, Malahat Kashfi-Moghadam

**Affiliations:** 1 University of Mohaghegh Ardabili, Department of Psychology, Ardabil 5619911367, Iran; 2 School of Health and Social Care, Teesside University, Middlesbrough TS1 3BA, UK; 3 Islamic Azad University science and research Branch, Tehran 1477893855, Iran

**Keywords:** fat percentage, inhibitory control, obesity, skeletal muscle mass, underweight, working memory

## Abstract

*Background and objectives:* To explore the association between weight status and executive function in young adults. *Materials and Methods:* Ninety-seven young males (age 17–26 years) underwent adiposity and body composition measurements using body composition analyzer. Inhibitory control and working memory were measured using the Cambridge Neuropsychological Test Battery (CANTAB). *Results:* Multiple linear regression using both unadjusted and adjusted analyses revealed no association between adiposity and body composition variables with executive tasks, apart from a significant association between skeletal muscle mass (SMM) and mean reaction time on go trial (standardized *B* = -0.28; *p* = 0.02). Multivariate analysis of covariance (MANCOVA) revealed that underweight participants presented inferior working memory compared to their normal weight (*p* = 0.001) or overweight peers (*p* = 0.008). However, according to the percentage fat quartiles (Q) participants with the highest quartile (Q4) were inferior in inhibitory control than their peers with Q2 (*p* = 0.04), and participants with the lowest quartile (Q1) were inferior in working memory compared with their peers with Q2 (*p* = 0.01) or Q3 (*p* = 0.02). A worse inhibitory control was observed for participants with the highest fat/SMM (Q4) compared to participants in Q3 (*p* = 0.03), and in contrast worse working memory was observed for participants with the lowest fat/SMM (Q1) compared to participants in Q2 (*p* = 0.04) or Q3 (*p* = 0.009). *Conclusions:* Low adiposity is associated with worse working memory, whereas high adiposity is associated with worse inhibitory control. Therefore, our findings show that normal adiposity, but greater SMM may have a positive impact on executive function in young adults.

## 1. Introduction

Overweight and obesity are associated with several health problems such as diabetes, hypertension, cardiovascular disease, as well as impaired cognitive performance [1,2,3]. The existing literature has frequently reported the association between obesity and impaired cognitive function in several domains in children and adolescents [2] and mid-life adults [3]. It has been suggested that neurological disorders in both the central and peripheral nervous systems are vulnerable to higher adiposity [4]. Likewise, cognitive impairment due to obesity might emerge later in life and during adulthood or old age [5], and therefore intervention programs to treat adults with obesity might attenuate the risk of dementia later in life [2,3].

However, methodological limitations of previous studies indicate the need for further studies in this area [2,3]. For instance, the effect of moderators on the association between obesity and cognitive function needs further exploring [2,3]. It is known that socioeconomic status (SES) is strongly associated with cognitive ability [6] and in some studies, SES, but not body mass index (BMI), has been associated with cognition [7]. Depression [8] and physical activity (PA) [9] have also been reported as other potential moderators. Another consideration is the type measurement of obesity applied, for instance, there is evidence that body fat percentage is a better predictor of overall obesity and metabolic risk factors than BMI [10], although most of the studies still use BMI as a measurement of obesity. Therefore, there is a need for high-quality studies using multiple valid and sensitive cognitive tests, including various covariates, and objective measures of adiposity.

On the other hand, not only overweight and obesity but also underweight is associated with health problems [1]. Nevertheless, most studies focused on the health effects of overweight and obesity and little information on the effect of underweight on health is available [11]. For instance, it has been recently reported that both lower body fat percentage and skeletal muscle mass (SMM) are associated with impaired cognitive function in older individuals [12,13,14], but there is no evidence available for the young populations.

To be successful in daily life, an individual needs self-control, creativity, discipline and flexibility. Executive functions are central to all those traits which make us able to give a considered instead of being impulsive when responding to a stimulus, mentally playing with ideas and staying focused [15]. Executive functions are not only important factors for learning and academic success in young people but also career and marriage in young adults [15].

Inhibitory control is a complex cognitive process which refers to the ability response but prevents an habitual response when needed. Working memory is the ability by which one retain information over the short term, monitor the relevance of incoming stimuli, and update information in memory when necessary [16]. Both inhibitory control and working memory are important aspects of core executive functions [15].

There are numerous studies underlying the association between obesity and executive functions such as inhibitory control and working memory in children, adolescents and middle-aged adults. However, findings are inconsistent [2,3]. More precisely, there is little evidence of these associations for special age groups such as young adults (18-35 years) when the brain has already reached its peak development.

Therefore, a better understanding how weight status compromises cognition a moderator analysis i.e. SES, depression, PA), on a range of weight groups (underweight, normal weight, overweight and obese), and use of objective measures of adiposity and body composition variables such as SMM, is needed. This might help to guide future obesity or learning intervention [2,3].

The aim of this study was to explore differences in inhibitory control and working memory in young adult males (17-26 years), from various weight status and body composition groups, taking into account known covariates such as age, SES, depressive symptoms, and PA. We hypothesized that both underweight and obesity are associated with worse inhibitory control and working memory in young adult males. We also hypothesize that higher SMM would be associated with better inhibitory control and working memory in young adult males.

## 2. Methods

### 2.1. Participants

Participants with various weight status were recruited using advertisements displayed in different settings including; public libraries, public high schools, and the University of Mohaghegh Ardabili, Ardabil, Iran. Due to sociocultural limitations, only male volunteers were recruited. Ninety-seven young males (age 17–26 years) participated in the present study. Only apparently healthy male participants were invited to participate. Exclusion criteria included the presence of musculoskeletal or neurological problems, chronic diseases (e.g. diabetes), head injury, taking medications or not interested in participating. Six participants were excluded from the study due to meet one or more of the exclusion criteria. The final sample included in the study analyses was 91 young males. All participants gave written informed consent, and the study was conducted in accordance with the Declaration of Helsinki. The protocol was approved by the Ethics Committee of Mohaghegh Ardabili University (Consent No. 001/2018 from 10 August 2018).

### 2.2. Procedures

Data was collected between August and October 2018 in the course of nine weeks in a physiological test lab at the University of Mohaghegh Ardabili. Each participant visited the lab twice. During the first visit (9 a.m to 2.30 p.m) participants were familiarized with the procedures of the study. Participants demographics and body composition data were recorded. Cognitive tasks were introduced to participants, and they were requested to avoid consumption of food and caffeine-containing beverages for 3-4 h, and to avoid vigorous PA at least 24 h before the cognitive measurements. On the second visit (24 h later from the first visit and from 9 a.m to 14:30 p.m) data collection of computerized cognitive tests were performed.

### 2.3. Anthropometry

Body mass, body fat percentage and SMM were acquired by using a X-Contact 356 body composition analyzer (Jawon Medical, Seoul, South Korea). The instrument with handlebar and footpad electrodes evaluates diverse body composition variables. Height was measured barefoot in the Frankfurt horizontal plane with a telescopic height measuring instrument (SECA 225; Chino, CA, USA) to the nearest 1 mm. BMI was calculated as body weight in kilograms divided by the square of height in meters (kg/m^2^).

### 2.4. Assessment of Executive Functions

Cognitive functions were assessed using the Cambridge Neuropsychological Test Battery (CANTAB) as a valid computerized program for measuring various cognitive tasks [17,18]. The CANTAB program was installed in a touch screen PC (ASUS, ET2012EUTS - 20.1 inches) for measuring Motor Screening Task (MST), Stop Signal Task (SST), Spatial Span (SSP), and Spatial Working Memory (SWM), which are described below. All the cognitive sessions, in addition to a 2–3 min break time between the tests lasted approximately 45 min.

#### 2.4.1. Motor Screening Test (MST)

The MST was performed to ensure that participants had no visual or comprehension problems. In the MST a pink cross appeared on a black touch screen and participants were asked to touch the center of the cross with their dominant hand.

#### 2.4.2. Stop Signal Task (SST)

Response inhibition was evaluated by using the SST, a unique version of a classic approach to measuring response inhibition. A two-choice button box was presented on the screen and participants were asked to respond to an arrow stimulus, by touching one of the two boxes depending on the direction in which the arrow points. In this regard, participants touched the right box on the touch screen as quick as possible with his index finger of the right hand when showed with a right-pointing arrow and vice versa for a left-pointing arrow with his index finger of the left hand. However, on 25% of trials, participants were presented with an auditory stop signal after showing the arrow (go signal). Participants were instructed to respond as quickly as possible except for those trials containing the auditory stop signal (a beep sound) which guided the participant to stop. Each participant completed five blocks, and a feedback screen was presented with the participants’ performance. The test administrator explained the feedback to the participants and encouraged them to perform the task as quick as possible. Measures included: the mean stop signal reaction time (SSRT) which is the mean time in which a person is able to inhibit the pre-potent response successfully; and the mean reaction time on go trials (Go RT) which is the mean time passed until a person press button on the screen when there is no stop signal.

#### 2.4.3. Spatial Working Memory (SWM)

The SWM provides a measure of strategy as well as errors and requires retention and manipulation of visuospatial information. During the trials, an increasing number of boxes appear on the screen. The test starts with two boxes and progresses to a maximum of nine boxes, and the test ends after two failed attempts. The participant was asked to look at the screen as a pattern of boxes emerges and to remember the pattern, and then repeat the pattern by touching the boxes. Participants were advised not to revisit a box that had already yielded a token. The analyzed measures were SWM-Between Errors (number of boxes revisited by a participant where a token box had previously been found) and SWM-strategy (number of boxes when participants began a new search by touching a different box, so, a high score shows an inefficient strategy).

#### 2.4.4. Spatial Span (SSP)

This test assesses working memory capacity. For completing SSP participant is required to remember and recall a sequential series of colored boxes in the correct forward order. The analyzed measures included: SSP-length (the longest sequences of boxes appear and remembered) and SSP-total errors (the number of times a wrong box is chosen).

### 2.5. Covariates

On the first visit, the following data were measured. Blood pressure [2,3] was measured using standard mercury sphygmomanometers (Model 1002; Presameter, Riester, Germany). This was measured three times and averaged to provide mean systolic and diastolic blood pressure. SES was computed from parents’ educational and occupational status, as explained in the previous study [19]. Participants were asked to fill the long form of the International Physical Activity Questionnaire (IPAQ). This is a standardized measure for PA which covers several domains of daily living PA, such as occupation, transportation, leisure time, household and family care [20]. The output of the IPAQ reports an estimation of total PA load in metabolic equivalent (MET)-min per week. The IPAQ has been shown to have acceptable validity and reliability in 18-65 years old adults [21]. The Beck Depression Inventory-II (BDI-II) was used as a reliable and valid measure of depressive symptoms [22].

### 2.6. Statistical Analyses

Data were checked for normality by using the Kolmogrove–Smirnove test and graphical models (e.g., boxplot, histogram, etc). All values except for total-PA showed normal distribution. Therefore, natural log transformation data of total-PA was applied when necessary.

Multiple linear regression analyses by using Enter method and both unadjusted and adjusted (i.e. age, blood pressure, SES, depressive symptoms, and total-PA) analyses were conducted between obesity or body composition variables as independent variables (BMI, %fat, SMM and fat mass to SMM ratio) and executive tasks as dependent variables (inhibitory control and working memory). To interpret the regression coefficient Cohen’s *f^2^* [23] was used (i.e., ≥0.02 = small, ≥0.15 = medium, ≥0.35 = large). Assuming a power of 0.80, and an α of 0.05, a sample of 91 participants is required for the regression analysis considering a medium effect size (*f*^2^ = 0.35) [24].

Unadjusted and adjusted (controlling for age, blood pressure, SES, depressive symptoms, and total-PA) multivariate analysis of covariance (MANCOVA) was applied. Analyses were conducted for comparison of executive functioning tasks across the weight status groups. BMI cut off points [25,26] defining weight status were used for categorizing the participants in one of the four groups, including underweight, normal weight, overweight and obese. Fat percentage, SMM and fat mass to SMM ratio (fat/SMM) were compared between quartiles (25, 50, 75 and 100 percentiles). Least significant difference (LSD) post hoc test was used for multiple comparisons. Cohen’s *d* (i.e., negligible for |*d*| < 0.2; small for 0.2 ≤ |*d*| < 0.5; medium for 0.5 ≤ |*d*| < 0.8; and large for |*d*| ≥ 0.8) and was calculated based on the partial η^2^ statistics to interpret the magnitude of the effect size [23]. Assuming a power of 0.80, and an α of 0.05, a sample of 91 is required for MANCOVA to explore the large effect size for analyses with four groups [24]. Results of Levene’s test of equality of error variance was found non-significant for all the dependent variables (*p* > 0.05). All calculations were performed using SPSS v.21.0 software for Windows (IBM, Armonk, NY, USA). Statistical significance was set at *p* ≤ 0.05

## 3. Results

### 3.1. General Characteristics

Demographic, physiological and physical characteristics as well as executive functioning tasks of the participants are shown in Table 1. The prevalence of underweight, normal weight, overweight and obesity according to the BMI cut off points [25,26] was 17.4% (*n* = 16), 45.3% (*n* = 41), 19.8% (*n* = 18), and 17.4% (*n* = 16), respectively.

### 3.2. Linear Approach

Results of multiple linear regression using both unadjusted and adjusted analyses revealed no association between adiposity and body composition variables with the underlying executive tasks, except for an association between SMM and Go RT (Unadj. standardized *B* = -0.24, *p* = 0.03; Unadj. standardized *B* = -0.24, *p* = 0.02; Table 2), which suggests that higher SMM is associated with a better Go RT in the young males. The observed effect sizes for the unadjusted and adjusted analyses were *f^2^* = 0.06 and *f^2^* = 0.10, respectively, which indicate a medium positive effect of SMM on GO RT.

### 3.3. Comparison Approach

**Figure 1:**
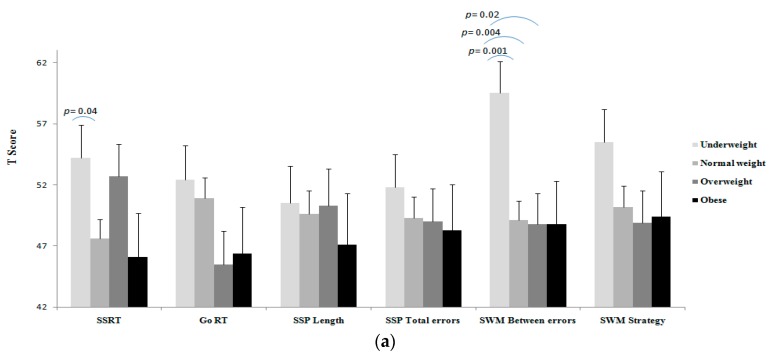

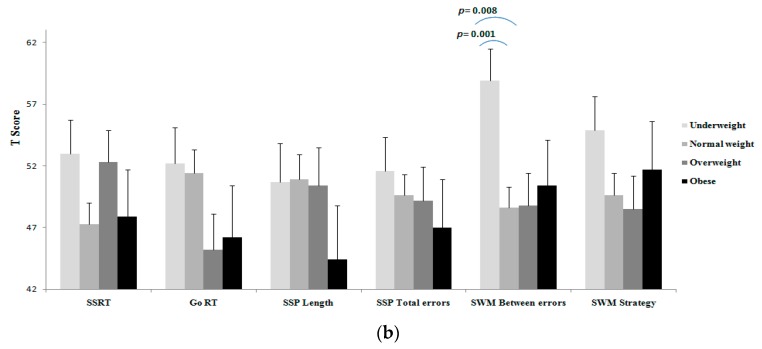
(**a**) Unadjusted comparison, (**b**) adjusted # comparison of executive function between the BMI categories. Natural log transformation data of total-PA was used as a covariate. Composite of *T* score of systolic and diastolic blood pressure was used as a covariate. #Values are reported after adjustment for age, SES, depressive symptoms, total PA, and blood pressure. Note: in all tasks (except for SSP length) lower score is better; *T* scores of the executive tasks were used for showing all in the same figure.

**Figure 2:**
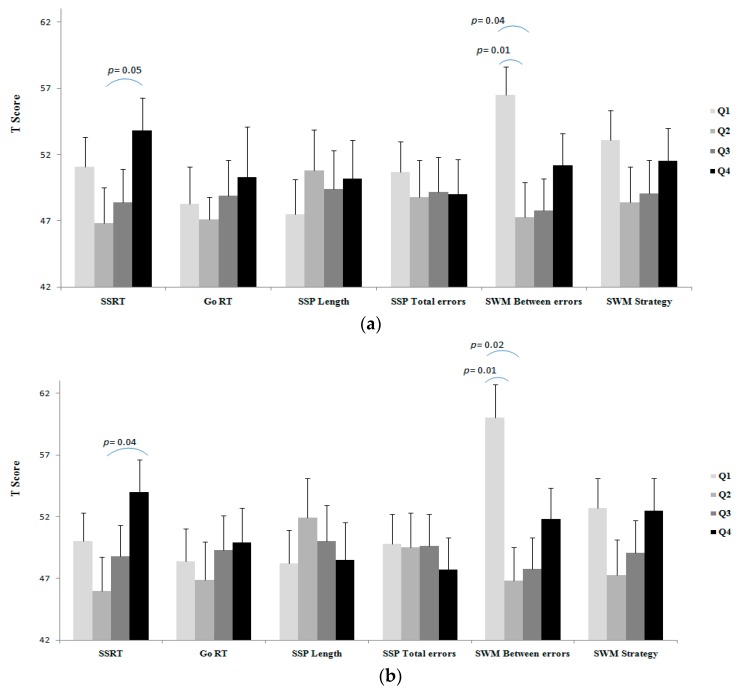
(**a**) Unadjusted comparison, (**b**) adjusted # comparison of executive function between the fat percentage quartiles. Composite of *T* score of systolic and diastolic blood pressure was used as a covariate. #Values are reported after adjustment for age, SES, depressive symptoms, total PA, and blood pressure. Note: in all tasks (except for SSP length) lower score is better; *T* scores of the executive tasks were used for showing all in the same figure.

**Figure 3:**
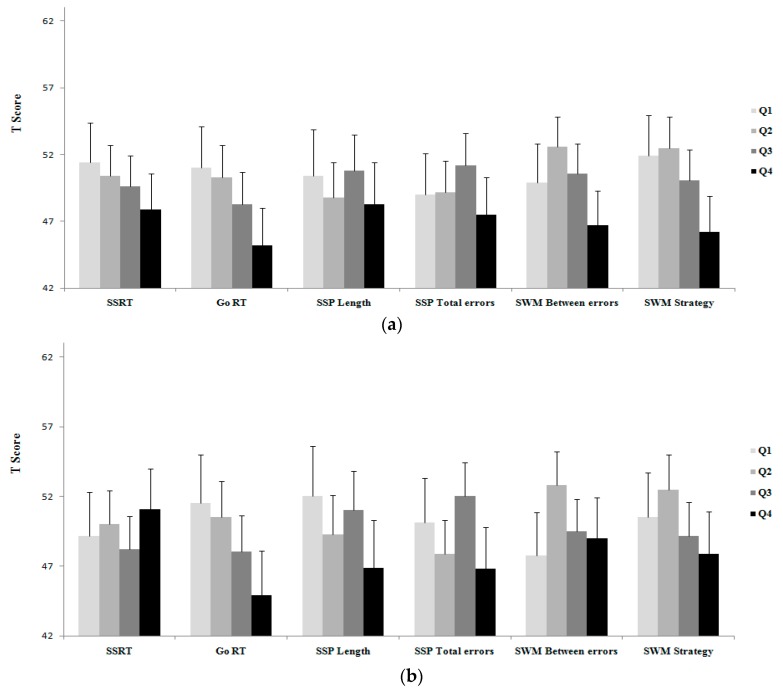
(**a**) Unadjusted comparison, (**b**) adjusted # comparison of executive function between the SMM quartiles. Natural log transformation data of total-PA was used as a covariate. Composite of *T* score of systolic and diastolic blood pressure was used as a covariate. #Values are reported after adjustment for age, SES, depressive symptoms, total PA, and blood pressure. Note: in all tasks (except for SSP length) lower score is better; *T* scores of the executive tasks were used for showing all in the same figure.

**Figure 4:**
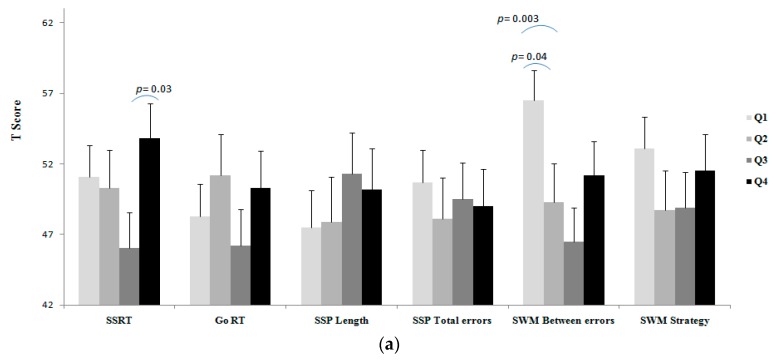

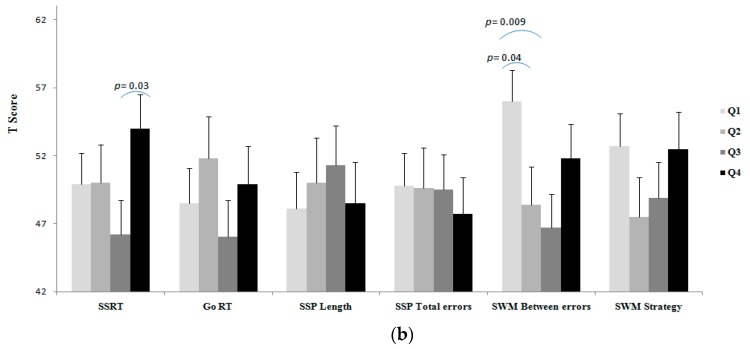
(**a**) Unadjusted comparison, (**b**) adjusted # comparison of executive function between the fat/SMM quartiles. Natural log transformation data of total-PA was used as a covariate. Composite of T score of systolic and diastolic blood pressure was used as a covariate. #Values are reported after adjustment for age, SES, depressive symptoms, total PA, and blood pressure. Note: in all tasks (except for SSP length) lower score is better; T scores of the executive tasks were used for showing all in the same figure.

Results of unadjusted and adjusted comparisons of executive functioning tasks between BMI categories, as well as fat percentage, SMM, and fat/SMM quartiles are shown in Figure 1, Figure 2, Figure 3 and Figure 4.

A worse SSRT for the underweight compared to the normal weight group was observed (Unadj. *p* = 0.04, *d* = 0.67, Figure 1a), but this disappeared after adjusting the analysis for the covariates (Figure 1b). The results of unadjusted analysis indicated a worse SWM-Between Errors for the underweight participants compared to the normal weight (*p* = 0.001), overweight (*p* = 0.004) and obese (*p* = 0.02) participants (Figure 1a; *d* = 95). In the adjusted analysis, however, a worse SWM-Between Errors was observed for the underweight participants compared to normal weight (*p* = 0.001) and overweight (*p* = 0.008) (Figure 1b; *d* = 94). This suggests a worse working memory for the underweight participants than the normal weight and overweight independent of the covariates. No differences were observed for GO RT, SSP-Length, SSP-Total Errors, and SWM-Strategy between the BMI categories in either unadjusted or adjusted analyses.

A worse SSRT was observed for the fat percentage Q4 group than the Q2 group (Unadj. *p* = 0.05; Figure 2a; Adj. *p* = 0.04; Figure 2b), which suggests that higher percentage of body fat is associated with a worse inhibitory control in the young males. The observed effect size for the unadjusted and adjusted analyses were *d* = 0.53 and *d* = 59, respectively. This indicates a medium negative impact of high fat percentage on inhibitory control when compared with the other weight status groups.

A worse SWM-Between Errors was observed for the percentage of body fat Q1 group compared to the Q2 (Unadj. *p* = 0.01, Figure 2a; Adj. *p* = 0.01, Figure 2b) and Q3 (Unadj. *p* = 0.04, Figure 2a; Adj. *p* = 0.02, Figure 2b) groups. The observed effect size for the unadjusted and adjusted analyses were *d* = 0.87 and *d* = 81, respectively. This indicates a strong negative impact of being underweight on working memory when compared with the other weight status groups. No differences were observed for GO RT, SSP Length, SSP-Total Errors and SWM-Strategy between the percentage fat quartiles in either unadjusted or adjusted analyses.

No differences were observed for the executive functioning tasks between the SMM quartiles in either unadjusted or adjusted analyses (Figure 3a,b).

A worse SSRT was observed for the fat/SMM Q4 group than the Q2 group (Unadj. *p* = 0.03; Figure 4a; Adj. *p* = 0.03; Figure 4b), which suggests that higher fat/SMM is associated with a worse inhibitory control in the young males. The observed effect size for the unadjusted and adjusted analyses were *d* = 0.59 and *d* = 61, respectively. This shows a medium negative impact of high fat/SMM on inhibitory control when compared with the other weight status groups.

A worse SWM-Between Errors was observed for the fat/SMM Q1 group compared to Q2 (Unadj. *p* = 0.04, Figure 4a; Adj. *p*= 0.04, Figure 4b) and Q3 (Unadj. *p* = 0.003, Figure 4a; Adj. *p* = 0.009, Figure 4b) groups. The observed effect size for the unadjusted and adjusted analyses were *d* = 0.85 and *d* = 81, respectively. This indicates a strong negative impact of lower fat/SMM on working memory when compared with the other groups. No differences were observed for GO RT, SSP-Length, SSP-Total Errors and SWM-Strategy between the fat/SMM quartiles in either unadjusted or adjusted analyses.

## 4. Discussion

The aim of the present study was to explore the association between weight status and executive function in young adult males. We hypothesized that both underweight and obesity are associated with worse underlying executive functioning tasks in young males independent of control for known covariates.

One of the limitations of previous studies which explored the association between weight status and cognitive function was using BMI as the only obesity index [27]. Instead, body fat percentage has been suggested as a better overall body obesity, predictor than BMI. This is because BMI as a measure of overall obesity does not account for varying proportions of fat, bone and muscle mass [10]. Furthermore, it has been shown that the association between body fat percentage and BMI is different among different ethnic groups [28], and body fat percentage is a better predictor of metabolic risk factors than BMI in children and adolescents [29].

The results of the linear approach performed in this study indicated no association between adiposity or body composition variables with the underlying executive tasks in both unadjusted or adjusted models, except for an association between SMM and Go RT. However, the results of the comparison approach were interesting as according to the BMI categories, underweight participants were inferior in working memory compared to their normal weight or overweight peers. Likewise, participants with the highest percentage of body fat were inferior in inhibitory control and participants in the lowest percentage of body fat quartiles were inferior in working memory than their peers in the normal percentage of body fat quartiles (Q2 or Q3). It is important to say that adjusting the analysis for the covariates had minimal effects on the association between weight status or body composition with executive function.

There is little evidence underlying the association between weight status and executive function in young adults (18-35 years) when the brain reaches a peak. In the middle-aged population, the association between obesity and inhibitory control is equivocal [3]. Some reported no association [30] while others reported a significant association [31,32]. In children and adolescent samples, however, numerous studies supported a negative association between obesity and inhibitory control [2], which agrees with the findings of the present study. However, the results of the association between obesity and working memory are inconsistent in the literature for both adolescents and middle-aged adults [16,32,33,34,35], and it seems that there is no evidence for an independent and reliable link between obesity and working memory [2,3], which coincides with findings of the present study.

Therefore, this current study reveals that according to both BMI and percentage fat categories both underweight (or the group in the lowest quartile of the percentage of body fat) and obesity (or the group with the highest quartile of percentage of body fat) were associated with worse executive function in the young adults independent of known covariates. However, the findings of impaired executive function in participants with underweight or with the lowest percentage fat might be impacted by medical or psychological conditions (eating-related disorders such as anorexia nervosa, etc.) not measured in this study. Furthermore, there is little evidence for the association between underweight and cognitive function in young people. However, in older adults, it was observed that underweight (but not overweight or obesity) is a risk factor for dementia and impaired cognitive function [12,13,14].

Most studies underlying the association between weight status and cognitive function in young (children, adolescents or young adults) and the middle-aged population did not consider simultaneous inclusion of both underweight and overweight/obese samples, likewise, most studies used only BMI as the obesity index measurement. Gunstad et al. [36], observed no association between BMI and cognitive function in a sample of children and adolescents. However, they suggested that underweight might be a risk factor for reduced memory performance. A recent study with almost two million people observed that underweight (measured by BMI) in middle age and old age carried an increased risk of dementia. Their results contradicted the hypothesis by which obesity in middle age could increase the risk of dementia [37]. Similarly, some recent studies have observed that underweight patients with anorexia nervosa had worse cognitive performance than their control peers [38]. Interestingly, during weight recovery, cognitive functioning was improved in those underweight adolescents [38].

Recent evidence shows that there is an association between the decreased SMM and percentage fat with impaired cognitive function independent of the potential of control for covariates such as sex, SES, smoking, etc. in older people [12,13,14]. In contrast, the evidence shows that both higher SMM and percentage of body fat seemed to impact positively in cognitive function in older adults, which was identified as the “obesity paradox” [13]. In the present study, although an association was observed between higher SMM and a better Go RT, no differences in any of the underlying executive performances were observed between SMM quartiles in the young males. Worse inhibitory control, however, was noted for participants in the highest fat/SMM quartiles, and in contrast, worse working memory was observed for the participants in the lowest fat/SMM quartile. These results are similar to the results observed for the percentage of fat quartiles and suggest that executive impairment is more affected with the highest or lowest percentage fat, rather than muscle mass in the young adult males. Furthermore, the results in most comparison models revealed a “U” shape underlying the association between adiposity and the executive tasks by which the young adult males with the lowest or highest percentage fat were inferior in inhibitory control or working memory than their peers. This may be the reason why linear approach did not reveal any association between obesity indices and underlying executive tasks, except between SMM with Go RT.

There are several possible mechanisms by which obesity or underweight impact cognitive function. For instance, both underweight and obesity are associated with brain changes [39,40], impaired glucose tolerance [41], and psychiatric disorders [11,42], which can impact negatively on cognitive function. Furthermore, insulin-like growth factor-I and brain-derived neurotrophic as another possible beneficial factor for cognitive function are decreased in both obesity and underweight subjects [43,44,45]. Finally, based on the findings of the present study and previous studies, the implication of better nutrition and physical activity to promote changes in weight status to a normal level and increase in SMM [27,38,46] might be an important strategy to impact positively cognitive function. However, to examine this, longitudinal and intervention studies are needed.

The study has some strengths including the use of objective methods for measuring percentage fat and SMM, including various obesity and body composition variables and participants in a range of weight status (underweight, normal weight or obesity). The study also used both linear and comparison approaches and controlled for known potential covariates such as age, SES, PA, blood pressure and depressive symptoms. However, the study has some limitations, including its cross-sectional nature and inclusion of only male participants. Therefore, longitudinal studies in both males and females are required to confirm these findings.

## 5. Conclusions

In summary, despite the limitations of the study, it was observed that high adiposity is associated with worse inhibitory control, and in contrast, low adiposity is associated with worse working memory. However, higher SMM seems to impact reaction time positively. Our findings suggest that a normal weight status is associated with a better executive function compared to unhealthy weight status (i.e. underweight and obesity) in the young males. However, longitudinal interventional studies are needed to examine whether interventions which promote positive changes in weight status to a normal level and increase in SMM is able to impact positively executive function in young adults in the longer term.

## Figures and Tables

**Table 1:** Demographics, physiological, and cognitive functioning characteristics of the participants (*n* = 91).

	Variables (unit)	Mean (SD)	Min–Max
Demographics & physiological characteristics	Age (year)	19.9 (3.5)	17.0–26.0
	SES	11.3 (4.3)	2.0–23.0
	Height (cm)	178.8 (6.5)	159.0–196.0
	Weight (kg)	75.3 (15.6)	52.0–120.0
	BMI (kg/m^2^)	23.5 (4.9)	16.6–38.1
	%Fat	16.8 (8.3)	3.1–35.4
	SMM (kg)	34.3 (4.3)	25.5–45.4
	Systolic blood pressure (mm Hg)	112.3 (13.3)	80.0–150.0
	Diastolic blood pressure (mm Hg)	72.4 (9.2)	50.0–110.0
	Total PA (MET-min/week)	7477.4 (5333.9)	150.0–19500.0
	BDI-II (score)	10.5 (7.0)	1.0–30.0
Stop signal task	Go RT (ms)	685.6 (163.7)	388.1–1125.9
	SSRT (ms)	229.4 (41.9)	140.3–361.0
SWM	Strategy (score)	31.3 (5.9)	19.0–43.0
	Between errors (score)	22.3 (17.3)	0.0–72.0
SSP	Span length (score)	6.9 (1.1)	4.0–9.0
	Total errors (score)	14.4 (6.6)	0.0–48.0

BDI-II: Beck Depression Inventory-II; SES: Socioeconomic status; SSRT: stop signal reaction time; SWM: spatial working memory; SSP: spatial span.

**Table 2:** Multiple linear regression analyses between adiposity and body composition variables with executive functioning tasks. Natural log transformation data of total-PA was used as covariate. Composite of *T* score of systolic and diastolic blood pressure was also used as a covariate. #Values are reported after adjustment for age, SES, depressive symptoms, total PA, and blood pressure. Note: the reported coefficients are standardized *B*.

		SSRT	Go RT	SSP Length	SSP Total Errors	SWM Between Errors	SWM Strategy
BMI	Unadj.	−0.07 (*p* = 0.54)	−0.13 (*p* = 0.22)	0.02 (*p* = 0.83)	−0.02 (*p* = 0.88)	−0.13 (*p* = 0.24)	−0.04 (*p* = 0.74)
	Adj. ^#^	0.00 (*p* = 0.00)	−0.19 (*p* = 0.14)	−0.09 (*p* = 0.48)	−0.01 (*p* = 0.93)	−0.08 (*p* = 0.55)	0.03 (*p* = 0.79)
%Fat	Unadj.	−0.03 (*p* = 0.76)	−0.04 (*p* = 0.72)	0.03 (*p* = 0.80)	−0.03 (*p* = 0.76)	−0.13 (*p* = 0.24)	−0.01 (*p* = 0.89)
	Adj. ^#^	0.03 (*p* = 0.80)	−0.06 (*p* = 0.62)	−0.06 (*p* = 0.60)	−0.03 (*p* = 0.81)	−0.09 (*p* = 0.48)	0.05 (*p* = 0.68)
SMM	Unadj.	−0.02 (*p* = 0.84)	−0.24 (*p* = 0.03)	0.01 (*p* = 0.89)	−0.01 (*p* = 0.92)	−0.10 (*p* = 0.36)	−0.11 (*p* = 0.32)
	Adj. ^#^	0.03 (*p* = 0.78)	−0.28 (*p* = 0.02)	−0.05 (*p* = 0.66)	−0.01 (*p* = 0.91)	−0.05 (*p* = 0.67)	−0.08 (*p* = 0.50)
Fat/SMM	Unadj.	−0.02 (*p* = 0.82)	−0.03 (*p* = 0.76)	0.03 (*p* = 0.75)	−0.01 (*p* = 0.96)	−0.11 (*p* = 0.32)	−0.01 (*p* = 0.94)
	Adj. ^#^	0.03 (*p* = 0.77)	−0.06 (*p* = 0.65)	−0.06 (*p* = 0.62)	0.01 (*p* = 0.97)	−0.06 (*p* = 0.60)	0.06 (*p* = 0.63)

SSRT: stop signal reaction time, SWM: spatial working memory, SSP: spatial span, SMM: Skeletal muscle mass.

## References

[1] Yanovski J.A. (2018). Trends in underweight and obesity: Scale of the problem. Nat. Rev. Endocrinol..

[2] Liang J., Matheson B.E., Kaye W.H., Boutelle K.N. (2014). Neurocognitive correlates of obesity and obesity-related behaviors in children and adolescents. Int. J. Obes. (Lond.).

[3] Prickett C., Brennan L., Stolwyk R. (2015). Examining the relationship between obesity and cognitive function: A systematic literature review. Obes. Res. Clin. Pract..

[4] O’Brien P.D., Hinder L.M., Callaghan B.C., Feldman E.L. (2017). Neurological consequences of obesity. Lancet Neurol..

[5] Wang J., Freire D., Knable L., Zhao W., Gong B., Mazzola P., Ho L., Levine S., Pasinetti G.M. (2015). Childhood and adolescent obesity and long-term cognitive consequences during aging. J. Comp. Neurol..

[6] Noble K.G., Norman M.F., Farah M.J. (2005). Neurocognitive correlates of socioeconomic status in kindergarten children. Dev. Sci..

[7] Baxter S.D., Guinn C.H., Tebbs J.M., Royer J.A. (2013). There is no relationship between academic achievement and body mass index among fourth-grade, predominantly African-American children. J. Acad. Nutr. Diet..

[8] Cserjesi R., Luminet O., Poncelet A.S., Lénárd L. (2009). Altered executive function in obesity. Exploration of the role of affective states on cognitive abilities. Appetite.

[9] Chang Y.K., Chu C.H., Chen F.T., Hung T.M., Etnier J.L. (2017). Combined effects of physical activity and obesity on cognitive function: Independent, overlapping, moderator, and mediator models. Sports Med..

[10] Nooyens A.C., Koppes L.L., Visscher T.L., Twisk J.W., Kemper H.C., Schuit A.J., Van Mechelen W., Seidell J.C. (2007). Adolescent skinfold thickness is a better predictor of high body fatness in adults than is body mass index: The Amsterdam growth and health longitudinal study. Am. J. Clin. Nutr..

[11] Mak K.K., Tan S.H. (2012). Underweight problems in Asian children and adolescents. Eur. J. Nucl. Med. Mol. Imaging.

[12] Bae S., Shimada H., Park H., Lee S., Makizako H., Doi T., Yoshida D., Tsutsumimoto K., Anan Y., Suzuki T. (2017). Association between body composition parameters and risk of mild cognitive impairment in older Japanese adults. Geriatr. Gerontol. Int..

[13] Noh H.M., Oh S., Song H.J., Lee E.Y., Jeong J.Y., Ryu O.H., Hong K.S., Kim D.H. (2017). Relationships between cognitive function and body composition among community-dwelling older adults: A cross-sectional study. BMC Geriatr..

[14] Nourhashémi F., Andrieu S., Gillette-Guyonnet S., Reynish E., Albarède J.L., Grandjean H., Vellas B. (2002). Is there a relationship between fat-free soft tissue mass and low cognitive function? Results from a study of 7105 women. J. Am. Geriatr. Soc..

[15] Diamond A., Lee K. (2011). Interventions shown to aid executive function development in children 4 to 12 years old. Science.

[16] Strauss E., Sherman E.M.S., Spreen O. (2006). A Compendium of Neuropsychological Tests: Administration, Norms and Commentary.

[17] Sahakian B.J., Owen A.M. (1992). Computerized assessment in neuropsychiatry using CANTAB: Discussion paper. J. R. Soc. Med..

[18] Smith P.J., Need A.C., Cirulli E.T., Chiba-Falek O., Attix D.K. (2013). A comparison of the Cambridge automated neuropsychological test battery (CANTAB) with “traditional” neuropsychological testing instruments. J. Clin. Exp. Neuropsychol..

[19] Moradi A., Esmaeilzadeh S. (2015). Association between reaction time, speed and agility in schoolboys. Sport Sci. Heal..

[20] (2005). The international physical activity questionnaire. http://www.ipaq.ki.se/.

[21] Craig C.L., Marshall A.L., Sjöström M., Bauman A.E., Booth M.L., Ainsworth B.E., Pratt M., Ekelund U., Yngve A., Sallis J.F. (2003). International physical activity questionnaire: 12-country reliability and validity. Med. Sci. Sports Exerc..

[22] Ghassemzadeh H., Mojtabai R., Karamghadiri N., Ebrahimkhani N. (2005). Psychometric properties of a Persian-language version of the Beck Depression Inventory–Second edition: BDI-II-PERSIAN. Depress. Anxiety.

[23] Cohen J. (1988). Statistical Power Analysis for the Behavioral Sciences.

[24] Faul F., Erdfelder E., Lang A.G., Buchner A. (2007). G*Power 3: A flexible statistical power analysis program for the social, behavioral, and biomedical sciences. Behav. Res. Methods.

[25] Cole T.J., Bellizzi M.C., Flegal K.M., Dietz W.H. (2000). Establishing a standard definition for child overweight and obesity worldwide: International survey. BMJ.

[26] Cole T.J., Flegal K.M., Nicholls D., Jackson A.A. (2005). Body mass index cut offs to define thinness in children and adolescents: International survey. BMJ.

[27] Golden R., Hassevoort K., Cannavale C., Edwards C., Thompson S., Burd N., Holscher H., Cohen N., Khan N. (2019). Lean body mass, but not fat mass, is associated with hippocampal memory performance (P14-011-19). Curr. Dev. Nutr..

[28] Deurenberg P., Yap M., Van Staveren W.A. (1998). Body mass index and percent body fat: A meta analysis among different ethnic groups. Int. J. Obes..

[29] Oliosa P.R., Zaniqueli D., Alvim R.D.O., Barbosa M.C.R., Mill J.G. (2019). Body fat percentage is better than indicators of weight status to identify children and adolescents with unfavorable lipid profile. Jornal de Pediatria (Versão em Português).

[30] Fagundo A.B., de-la-Torre R., Jiménez-Murcia S., Agüera Z., Granero R., Tárrega S., Botella C., Baños R., Fernández-Real J.M., Rodríguez R. (2012). Executive functions profile in extreme eating/weight conditions: From anorexia nervosa to obesity. PLoS ONE.

[31] Nederkoorn C., Smulders F.T.Y., Havermans R.C., Roefs A., Jansen A. (2006). Impulsivity in obese women. Appetite.

[32] Ariza M., Garolera M., Jurado M.A., Garcia-Garcia I., Hernan I., Sánchez-Garre C., Vernet-Vernet M., Sender-Palacios M.J., Marques-Iturria I., Pueyo R. (2012). Dopamine genes (*DRD2/ANKK1-TaqA1* and *DRD4-7R*) and executive function: Their interaction with obesity. PLoS ONE.

[33] Gonzales M.M., Tarumi T., Miles S.C., Tanaka H., Shah F., Haley A.P. (2010). Insulin sensitivity as a mediator of the relationship between BMI and working memory-related brain activation. Obesisty.

[34] Verdejo-García A., Pérez-Expósito M., Schmidt-Río-Valle J., Fernández-Serrano M.J., Cruz F., Pérez-García M., López-Belmonte G., Martin-Matillas M., Martin-Lagos J.A., Marcos A. (2010). Selective alterations within executive functions in adolescents with excess weight. Obesisty.

[35] Maayan L., Hoogendoorn C., Sweat V., Convit A. (2011). Disinhibited eating in obese adolescents is associated with orbitofrontal volume reductions and executive dysfunction. Obesisty.

[36] Gunstad J., Spitznagel M.B., Paul R.H., Cohen R.A., Kohn M., Luyster F.S., Clark R., Williams L.M., Gordon E. (2008). Body mass index and neuropsychological function in healthy children and adolescents. Appetite.

[37] Qizilbash N., Gregson J., Johnson M.E., Pearce N., Douglas I., Wing K., Evans S.J.W., Pocock S.J. (2015). BMI and risk of dementia in two million people over two decades: A retrospective cohort study. Lancet Diabetes Endocrinol..

[38] Lozano-Serra E., Andrés-Perpiñá S., Lázaro-García L., Castro-Fornieles J. (2014). Adolescent anorexia nervosa: Cognitive performance after weight recovery. J. Psychosom. Res..

[39] Ou X., Andres A., Pivik R., Cleves M.A., Badger T.M. (2015). Brain gray and white matter differences in healthy normal weight and obese children. J. Magn. Reson. Imaging.

[40] Seitz J., Herpertz-Dahlmann B., Konrad K. (2016). Brain morphological changes in adolescent and adult patients with anorexia nervosa. J. Neural Transm..

[41] Jauch-Chara K., Schmoller A., Oltmanns K.M. (2011). Impaired glucose tolerance in healthy men with low body weight. Nutr. J..

[42] Petry N.M., Barry D., Pietrzak R.H., Wagner J.A. (2008). Overweight and obesistyity are associated with psychiatric disorders: Results from the national epidemiologic survey on alcohol and related conditions. Psychosom. Med..

[43] Okereke O., Kang J.H., Ma J., Hankinson S.E., Pollak M.N., Grodstein F. (2007). Plasma IGF-I levels and cognitive performance in older women. Neurobiol. Aging.

[44] Schneider H.J., Saller B., Klotsche J., März W., Erwa W., Wittchen H.U., Stalla G.K. (2006). Opposite associations of age-dependent insulin-like growth factor-I standard deviation scores with nutritional state in normal weight and obese subjects. Eur. J. Endocrinol..

[45] Monteleone P., Fabrazzo M., Martiadis V., Serritella C., Pannuto M., Maj M. (2005). Circulating brain-derived neurotrophic factor is decreased in women with anorexia and bulimia nervosa but not in women with binge-eating disorder: Relationships to co-morbid depression, psychopathology and hormonal variables. Psychol. Med..

[46] McLean F.H., Grant C., Morris A.C., Horgan G.W., Polanski A.J., Allan K., Campbell F.M., Langston R.F., Williams L.M. (2018). Rapid and reversible impairment of episodic memory by a high-fat diet in mice. Sci. Rep..

